# Protocol for imaging-based quantification of RNAPII clearance during transcription-coupled DNA repair

**DOI:** 10.1016/j.xpro.2025.104213

**Published:** 2025-11-20

**Authors:** Bram A.F.J. de Groot, Paula J. van der Meer, Diana van den Heuvel, Martijn S. Luijsterburg

**Affiliations:** 1 Leiden University Medical Center (LUMC), Department of Human Genetics, Einthovenweg 20, 2333 ZC Leiden, the Netherlands

**Keywords:** Cell Biology, Cell culture, Cell-based Assays, Microscopy

## Abstract

Elongating RNA polymerase II (RNAPII) stalls at transcription-blocking lesions in the DNA template strand and is removed by transcription-coupled DNA repair (TCR) factors. Here, we present a protocol to measure RNAPII clearance during TCR at sites of localized UV-induced DNA damage in adherent cells. We describe how to induce local UV damage and visualize the damaged area and chromatin-bound RNAPII levels using immunofluorescence staining. This approach can quantify the clearance of DNA damage-stalled RNAPII and its dependence on TCR factors.

For complete details on the use and execution of this protocol, please refer to van den Heuvel et al.^1^

## Highlights

• Protocol for tracking RNAPII clearance at UV damage sites via IF staining
• Steps for IF staining of local UV-induced damages and RNAPII
• Guidance on UV-C damage induction and DRB treatment to track elongating RNAPII
• Procedures for imaging and quantifying RNAPII clearance using ImageJ

## Abstract (editor highlights)

Publisher’s note: Undertaking any experimental protocol requires adherence to local institutional guidelines for laboratory safety and ethics.

## Graphical abstract

**Figure:**
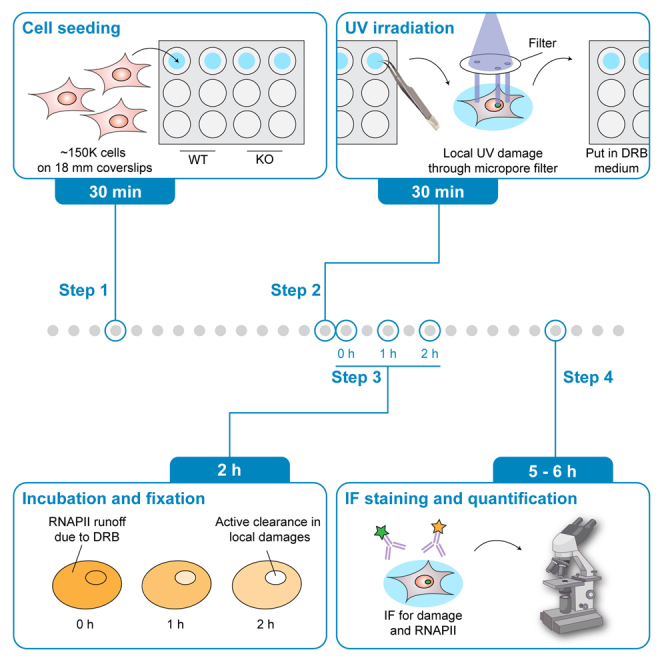

## Before you begin

Removal of RNA polymerase II (RNAPII) from DNA after stalling at a transcription-blocking DNA lesion is a crucial step in transcription-coupled repair (TCR). The TCR pathway is initiated when transcribing RNAPII stalls at DNA damage in the template strand, such as cyclobutane pyrimidine dimers (CPDs) caused by ultraviolet (UV) light exposure.^2^^,^^3^ Initially, the sequential and cooperative actions of TCR proteins CSB, CSA, ELOF1, UVSSA and STK19 recruit and position TFIIH onto DNA damage-stalled RNAPII.^1^^,^^4^^,^^5^^,^^6^^,^^7^ The subsequent clearance of RNAPII is required to provide downstream nucleotide excision repair (NER) proteins access to the lesions, enabling dual incision and ultimately repair of the damaged DNA.^8^^,^^9^^,^^10^

### Innovation

**Timing: 0.5–1 h**

**Figure 1:**
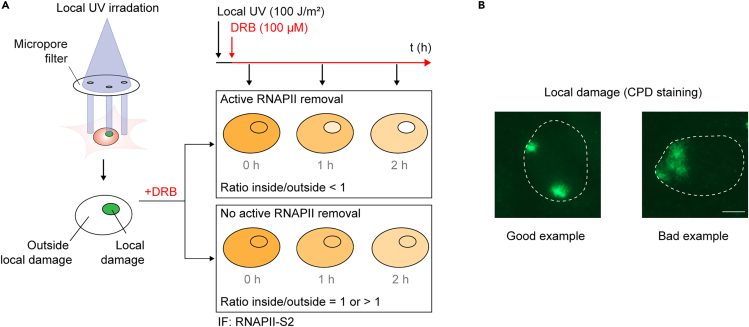
Experimental setup, principle, and expected RNAPII clearance outcome (A) Graphical representation of the RNAPII clearance in TCR proficient and deficient cells. The yellow color in and outside the damaged area represents the elongating RNAPII (RNAPII) intensity after IF. To calculate the rate of clearance the signal inside the damaged area is divided over outside. (B) Representative cell showing clear and bright CPD damage spots (good example left). Cell showing a vague and large (low intensity and overlaying a large part of the nucleus) CPD damage spot (bad example right). The scale bar represents ∼5 μm.

Our approach enables the measurement and quantification of RNAPII clearance from sites of local UV-induced DNA damage. Despite the importance of this step in TCR, an accessible experimental system to measure RNAPII clearance from damaged DNA has been lacking. To address this need, we recently developed an imaging-based approach to measure RNAPII clearance, which we previously used to demonstrate that STK19 is required for efficient RNAPII removal.^1^ Similarly, the absence of other core TCR factors, specifically CSB and CSA, impairs RNAPII clearance from sites of DNA damage, resulting in highly toxic, persistently stalled RNAPII complexes in the genome. Our experimental approach is immunofluorescence-based, and, while demonstrated here using RPE1 cells, it can be applied to any adherent cell type suitable for immunofluorescence (IF) staining and microscopy. In this protocol, cells are seeded on coverslips in a 12-well format, starved in medium with low serum, and then subjected to UV-C irradiation through a 5 μm micropore filter. This generates, on average, one localized area of DNA damage per nucleus, while the rest of the nucleus remains undamaged. Immediately after UV treatment, cells are incubated in medium containing 5,6-dichloro-1-β-D-ribofuranosylbenzimidazole (DRB), which prevents the release of new RNAPII molecules into elongation. This ensures that only RNAPII molecules already engaged in elongation at the time of damage induction are monitored.^11^ Cells are fixed at 0, 1 or 2 hours post-treatment, and RNAPII levels in both damaged and undamaged regions are visualized by IF staining using antibodies against CPDs to mark damaged areas and against elongating (Ser2-phosphorylated) RNAPII (Figure 1A). We provide guidelines and practical tips for performing microscopic analysis and downstream image quantification using ImageJ to assess RNAPII clearance efficiency during TCR.

Before beginning this protocol, take the following steps into account.

1. Set up a TUV PL-S 9W UV-C lamp (Philips) with a wavelength of 254 nm to irradiate cells using 100 J/m^2^ (joules per square meter) in order to induce localized UV-induced DNA damage.

**CRITICAL:** Ensure that the UV-C lamp is positioned inside a protective enclosure, in accordance with local safety guidelines, and arranged so that cells can be safely exposed to a precise dose of UV-C light by controlling the exposure time.

2. Measure the dose rate emitted by the installed UV-C lamp using a UV dosimeter IL1400BL SEL240-NS254-W (International Light Technologies) or equivalent.
  ***Note:*** The dose rate is measured in W/m^2^ (equivalent to J/m^2^/s).
  a. Use the following formula to calculate the exposure time required to deliver a dose of 100 J/m^2^.
    $$Exposure\;time\;\left(s\right)=\frac{Required\;radiation\;dose\;\left(J/m^{2}\right)}{Measured\;dose\;rate\;\left(J/m^{2}/s\right)}$$
3. Use cells that can adhere to uncoated glass coverslips, allowing them to be removed from the culture medium for localized UV-C irradiation, followed by IF staining and imaging.

***Note:*** If cells poorly adhere to uncoated glass, coverslips coated with fibronectin or poly Lysine can be used to prevent cells from detaching during this protocol.

***Note:*** This protocol has been applied to U2OS and primary fibroblast as an alternative to RPE1 cells (data not shown).

4. Ensure access to a fluorescence microscope, such as a Zeiss Axio Imager M2 used in our set-up.^1^^,^^12^
5. Prepare complete DMEM culture medium by adding 45 mL (8%) of fetal calf serum (FCS) and 5 mL (1%) of 100× penicillin-streptomycin (p/s) to one 500 mL bottle of DMEM GlutaMAX.
6. Prepare starvation medium by adding 5 mL (1%) of 100× penicillin-streptomycin (p/s) to one 500 mL bottle of DMEM GlutaMAX without adding FCS.
7. Culture cells at 37°C with 5% CO_2_ for at least two passages to eliminate residual stress from thawing.
8. Ensure that the coverslips are sterile before seeding, for example by sterilizing in an autoclave.

## Key resources table

**Table:** 

REAGENT or RESOURCE	SOURCE	IDENTIFIER
**Antibodies**		

DAPI (4,6-diamidino-2-phenylindole)	Thermo Fisher Scientific	D1306
Goat anti-mouse Alexa 488 antibody	Thermo Fisher Scientific	Cat#A-11029
Goat anti-rabbit Alexa 555 antibody	Thermo Fisher Scientific	Cat# A-21429
Mouse anti-CPD antibody	Cosmo Bio	Cat#CAC-NM-DND-001
Rabbit anti-RNAPII-S2 antibody	Abcam	Cat#ab5095

**Chemicals, peptides, and recombinant proteins**		

5,6-dichlorobenzimide 1-ß-D-ribofuranoside (DRB)	Sigma	Cat#D1916-50MG
Bovine serum albumin (BSA) powder	Sigma	Cat# 10735086001
DMEM GlutaMAX	Gibco	Cat#31966-047
Fetal calf serum (FCS)	Biowest	s1600-500
Formaldehyde 37% (v/v)	Sigma	Cat#252549-1L
Glycine powder	Sigma	Cat# G7126-5KG
NaOH powder	Merck	Cat#106498
Penicillin/streptomycin (p/s) 100×	Sigma	P4333
Phosphate-buffered saline (PBS)	Made in-house	Made in-house
Sterile PBS	Thermo Fisher Scientific	10010023
Triton X-100	Sigma	CAS# 9036-19-5
Trypsin solution	Made in-house	Made in-house
Tween 20	Sigma	Cat# P1379-500ML

**Deposited data**		

Raw and analyzed data	This paper	Source data

**Experimental models: Cell lines**		

RPE1-hTERT (Cas9i, PAC/TP53-dKO), CSA-KO (clone 3-1)	Van der Weegen et al.^6^	https://doi.org/10.1038/s41556-021-00688-9
RPE1-hTERT (Cas9i, PAC/TP53-dKO), CSB-KO (clone 15)	Van der Weegen et al.^6^	https://doi.org/10.1038/s41556-021-00688-9
RPE1-hTERT (Cas9i, PAC/TP53-dKO), parental	Van der Weegen et al.^6^	https://doi.org/10.1038/s41556-021-00688-9

**Software and algorithms**		

ImageJ (preferably v.1.48 or up)	Schneider et al.^13^	https://imagej.net/ij/https://doi.org/10.1038/nmeth.2089
ImageJ plugin “bioformats_package.jar”	Schneider et al.^13^	https://www.openmicroscopy.org/bio-formats/downloads
In-house ImageJ macro “RNAPII_clearance_DataExtraction_ImageJ_DvdH_20250905_final.txt”	This manuscript. Macro is an updated version from Van der Meer et al.^12^	https://git.lumc.nl/dvandenheuvel/ggr-uds-protocol_lumc_luijsterburglab https://git.lumc.nl/afjdegroot/rnapii-clearance
In-house data processing Excel sheet “DRB_runoff_local_UV_worksheet_v11_template.xlsx”	This manuscript	https://git.lumc.nl/afjdegroot/rnapii-clearance
Zen (2012 Blue edition)	Zeiss	https://www.zeiss.com/microscopy/en/products/software/zeiss-zen.html

**Other**		

12-well plates	Costar	3512
15 cm culture dishes	Sarstedt	833.903
Aqua poly/mount	Brunschwig	18606
Cell culture incubator (5% CO_2_, 37°C)	Thermo Fisher Scientific	Hera cell 150
Coverslips 18 mm	VWR	631-1580
DMEM, high glucose, GlutaMAX supplement, pyruvate	Gibco	31966-047
Flow cabinet	Clean air	EN12469
Fluorescence microscope	Zeiss	Zeiss Axio Imager M2
International Light NIST traceable radiometer photometer	NIST	IL1400BL SEL240-NS254-W
Isopore 5 μm filters	Milipore	Cat# TMTP04700
Microscope sides	Epredia Superfrost ground 45° white tab	AB00008032E01MNZ10
Parafilm	Parafilm M	Pm996
TUV PL-S 9 W UV-C lamp 254 nm	Philips	N/A
Tweezers curved	Sigma	Cat#Z168785
Falcon tube 50 mL	Greiner CELLSTAR	227261-N
Safe-lock Tubes 1.5 mL	Eppendorf	O220619J
25 mL serological pipette	Greiner	LOT: F2412335
10 mL serological pipette	Greiner	LOT: F2503354
PIPETMAN	Rainin	Pipet-Lite XLS
PIPETBOY	Integra	Acu2
Timer Traceable	VWR	# 62344-912
Immersion oil	Merck	104699

## Materials and equipment

***Alternatives:*** In this protocol, the Zeiss Axio Imager M2 fluorescence microscope is used to image RNAPII and CPD intensities. However, any fluorescence microscope is suitable, provided it can image at least three separate fluorescence channels and accommodate fixed cells on microscopy slides. Furthermore, this protocol uses a TUV PL-S 9W UV-C lamp (Philips) 254 nm, but any other UV-C lamp may be used, as long as it can deliver a dose of 100 J/m^2^ before the cells dry out (approximately 2 min).

**Table:** 

Name	Reagents
DRB medium^a^	50 μL DRB (5,6-dichloro-1-β-D-ribofuranosylbenzimidazole) 100 mM, 50 mL DMEM + p/s, Total volume 50 mL
Formaldehyde 3.7% solution	5 mL formaldehyde 37% (v/v), 45 mL PBS, Total volume 50 mL
0.5% Triton-X solution	250 μL Triton-X (100% v/v), 50 mL PBS, Total volume 50 mL
3% BSA solution	1.5 g BSA powder, 50 mL PBS, Total volume 50 mL
10% BSA solution	5 g BSA powder, 50 mL PBS, Total volume 50 mL
100 mM glycine solution	375 mg glycine powder, 50 mL PBS, Total volume 50 mL
0.5 M NaOH solution	20 g NaOH powder, 1000 mL H_2_O (Milli Q), Total volume 1000 mL
Washing Buffer (WB)	250 mg BSA powder, 250 μL Tween 20 (100% v/v), 50 mL PBS, Total volume 50 mL
Primary antibody solution	1 μL mouse anti-CPD (1/1000), 1 μL rabbit anti-RNAPII-S2 (1/1000), 998 μL Washing buffer (WB), Total volume 1 mL
Secondary antibody solution	1 μL goat anti-mouse Alexa 488 (1/1000), 1 μL goat anti-rabbit Alexa 555 (1/1000), 1 μL DAPI (1 mg/mL) solution 1/1000 (final concentration: 1 μg/mL), 997 μL Washing buffer (WB), Total volume 1 mL

a DRB medium: Always make the solution fresh.

**CRITICAL:** DRB is a toxic chemical. Use gloves, wear protective clothing.

**Formaldehyde 3.7% solution:** Store at 4°C for up to 1 month.

**CRITICAL:** Formaldehyde is a carcinogen and is toxic. Use gloves, wear protective clothing, and work in a fume hood to prevent exposure.

**0.5% Triton-X solution:** Store at 4°C for up to 3 months.

**3% BSA solution:** Store at 4°C for up to 2 weeks.

**10% BSA solution:** Store at 4°C for up to 2 weeks.

**100 mM Glycine solution:** Store at 4°C for up to 2 weeks.

**0.5 M NaOH solution:** Store at 4°C for up to 6 months.

**CRITICAL:** NaOH is a corrosive chemical. Use gloves, wear protective clothing, and work in a fume hood to prevent exposure.

**Washing Buffer (WB):** Store at 4°C for up to 2 weeks.

**Primary antibody solution:** Make freshly on the day of the experiment, keep at 18°C–22°C before use.

**Secondary antibody solution:** Make freshly on the day of the experiment, keep at 18°C–22°C in the dark before use.

### Software

• Imaging software: Zen (2012 Blue edition).
• Data analyses software: ImageJ (version 1.48), with the following requirements:
• ImageJ Plugin “bioformats_package.jar” at https://www.openmicroscopy.org/bio-formats/downloads.
• In-house generated ImageJ macro “RNAPII_clearance_DataExtraction_ImageJ_DvdH_20250905_final.txt” available at https://git.lumc.nl/afjdegroot/rnapii-clearance.
• In-house generated Excel quantification file “DRB_runoff_local_UV_worksheet_v11_template.xlsx”. The Excel sheet, example data (9 pictures of replicate 1 (first picture of each time-point and condition), and a filled-in Excel sheet with data of replicate 1 is also available at https://git.lumc.nl/afjdegroot/rnapii-clearance.

## Step-by-step method details

### Day 1—Cell seeding

**Timing: 30–45 min**

Objective: seed cells to reach ∼90% confluency for the UV irradiation on day 3.

1. Seed 150.000 RPE1 cells per well in a 12-wells plate, placing them on top of a sterile coverslip. For each condition being tested for RNAPII clearance, seed three time points (0 h, 1 h and 2 h after UV) in duplicate, resulting in six wells per condition. Refer to the graphical abstract and Figure 1A for an overview of the experimental setup.
  ***Note:*** It is convenient, but not strictly necessary, to place coverslips belonging to each time point in a separate 12-well plate during UV irradiation and fixation.
  **CRITICAL:** A 0 h control should be included to determine the intensity of RNAPII directly after UV irradiation. In addition, a TCR-proficient cell line, such as RPE1-hTERT WT cells, should be included to determine the level of RNAPII clearance in native conditions.
  a. Wash cells in a semi-confluent T75 flask with sterile PBS.
  b. Add 1 mL trypsin to the flask.
  c. Place the cells in a cell culture incubator at 37°C for 2–4 minutes, until cells detach.
  d. Inactivate trypsin by resuspending the cells in 9 mL DMEM (+p/s, FBS 8%).
  e. Count the cells and seed 150.000 cells in a well containing a total of 1 mL DMEM (+p/s, FCS 8%).
  f. Incubate overnight at 37°C with 5% CO_2_.

### Day 2—Medium starvation

**Timing: 15 min**

Objective: reduce the number of dividing cells to prevent replication from interfering with RNAPII clearance by TCR.

2. Replace the regular medium with starvation medium (DMEM with p/s, but without FCS).
  a. Remove the regular medium.
  b. Wash the cells with 1 mL starvation medium.
  c. Add 1 mL starvation medium to the cells.
3. Incubate the cells overnight at 37°C with 5% CO_2_.

### Day 3—Local UV-C radiation and fixation of cells

**Timing: 3 h**

Objective: locally irradiate cells with UV-C light through a micropore filter to induce, on average, one localized site of DNA damage per nucleus, followed by fixation at 0, 1 or 2 h post-irradiation.

4. Turn on the UV-C lamp.

***Note:*** Allow the UV-C lamp to warm up for approximately 10 min before use to ensure accurate and consistent UV-C dosing.

5. Before UV treatment, prepare a new 12-well plate with the same number of well as used in the experiment.
  a. Add 1 mL of starvation medium supplemented with 100 μM DRB to each well. Store the prepared 12-well plate at 37°C until you are ready for UV treatment.

**CRITICAL:** Ensure that each well contains enough medium to fully submerge the coverslip in the next steps.

6. Prepare 1 mL per well of 3.7% (v/v) formaldehyde in PBS to fix the cells after UV treatment.
7. Collect the following materials for local UV-C irradiation:
  a. A platform for irradiation, such as the lid of a 15 cm culture dish covered with parafilm.
  b. The 12-well plates containing 1 mL starvation medium supplemented with 100 μM DRB per well (from step 5).
  c. Tweezers.
  d. A timer.
  e. 5 μm micropore filters.
  f. 50 mL PBS in a falcon tube, and an empty 15 cm dish for rinsing filters.
8. Remove the medium from the cells and add 1 mL of PBS per well.
9. Bring the cell plates and materials (from step 7) to the UV-C lamp. Perform steps 10 to 12 near the lamp.
10. Rinse the required number of 5 μm filters on both sides using PBS and tweezers. Use the empty 15 cm dish for this.

***Note:*** Filters can be reused for up to three rounds of UV-C radiation treatment.

11. UV irradiate the cells and directly place them in DRB medium:
  a. Using tweezers, take the coverslips from the PBS and put them on the 15 cm dish lid covered with parafilm.
    ***Note:*** Place the two duplicate coverslips close together to fit both under one micropore filter to reduce the amount of filters necessary.
    **CRITICAL:** Place the coverslips with the cells facing up and do not let the cells stay dry for too long (maximum of 2 min until UV-C treatment). Avoid mixing up coverslips during this step.
  b. Cover every two duplicate coverslips with a rinsed 5 μm filter.
    **CRITICAL:** Let any remaining PBS fall off the filter before and use some pressure when applying the filter to minimalize the space between the filter and the coverslip. Residual drops of PBS on top of the filter or air bubbles between the filter and the cells will reduce the UV-C dose.
  c. Irradiate the coverslips through the micropore filters with 100 J/m^2^.
    ***Note:*** Start a timer to keep track of the incubation times after irradiation.
    ***Note:*** The thickness of the of the micropore membranes is 20 μm and the distance from the lamp to the coverslips is ∼1 m.
  d. Remove the filters carefully using tweezers.
    ***Note:*** Add a few drops of PBS on top of the micropore filters to remove them more easily.
  e. Put the coverslips in the wells of the 12-well plate containing freshly prepared starvation medium supplemented with 100 μM DRB.
    **CRITICAL:** Avoid mixing up coverslips during this step.
  f. Fix the 0 h time point directly after irradiation with UV:
    i. Remove the medium and wash with 1 mL of PBS.
    ii. Fix with 1 mL of 3.7% formaldehyde solution. Incubate at 18°C–22°C for 15 minutes.
    iii. Wash the cells 3 × 5 minutes with 1 mL of PBS.
    iv. Store the plate(s) in the fridge with 1 mL of PBS in each well until IF staining.
  g. Put the plates with the 1 h and 2 h timepoints in the incubator at 37°C and 5% CO_2_ and incubate for the corresponding times.
  h. Fix the remaining 1 hour and 2 hour timepoints after their corresponding incubation times as described previously in step 11f.
    ***Note:*** After fixing, store the cells at 4°C for up to 5 days until IF staining, or immediately continue with step 12.
    **Pause point:** Store up till 5 days at 4°C until IF.

### Day 4—Immunofluorescence staining of local damages and RNAPII

**Timing: 4–5 h**

Objective: Perform immunostaining on the cells with an antibody against CPD lesions to visualize the damaged areas in the nuclei, and against elongating RNAPII to visualize the transcribing RNAPII inside and outside the damaged areas. Remove the solutions after each washing, blocking and staining step using a pipet or a suction system. All steps are performed at 18°C–22°C unless stated differently.

12. Remove the PBS from the coverslips.
13. Permeabilize the cells with 1 mL of 0.5% Triton-X for 15 minutes.
14. Wash 2 × 5 minutes with 1 mL of 3% BSA in PBS to block the coverslips.

***Note:*** BSA is added before glycine treatment to primarily block the coverslips and reduce non-specific background.

15. Wash 1 × 5 minutes in 1 mL PBS.
16. Treat the coverslips with 100 mM glycine in PBS for 10 minutes.

***Note:*** Glycine is used to block unreacted aldehydes from the formaldehyde treatment.

17. Wash the cells with 1 mL of PBS for 5 minutes.
18. Denature the DNA with 1 mL of 0.5 M NaOH in PBS for 5 minutes. Remove the NaOH directly after 5 minutes.

***Note:*** Denaturing the DNA is required for the CPD antibody to bind the lesions.

**CRITICAL:** Longer incubation with NaOH can lead to fragmentation of the DNA.

19. Wash the cells 2 × 5 minutes with 1 mL PBS to remove the residual NaOH.
20. Reduce the volume needed in the following washing and blocking steps of the protocol to approximately 0.3 mL per coverslip (unless stated differently).
  a. Move the coverslips from the wells to a parafilm-covered surface using tweezers.

***Note:*** Preferentially, move the coverslips to box that can keep them in the dark for the secondary antibody staining in step 25. Drop PBS on the coverslips after moving them to avoid drying the cells.

**CRITICAL:** Mark the parafilm to avoid mixing up coverslips during this step.

21. Block the coverslips with 10% BSA in PBS for 15 minutes.

***Note:*** The second blocking step is performed to ensure that NaOH used to denature DNA in step 18 is fully neutralized and does not denature the primary antibody used in step 23.

22. Equilibrate the coverslips with washing buffer (WB) for 15 minutes.
23. Incubate the coverslips with 100 μL primary antibody solution to stain for CPDs and RNAPII. Incubate for 2 h at 18°C–22°C or overnight at 4°C.
24. Wash the cells 4 × 5 minutes with WB.
25. Incubate the coverslips with 100 μL secondary antibody solution supplemented with DAPI 1 h in the dark.

**CRITICAL:** The secondary antibodies and DAPI are light sensitive. Keep the coverslips in the dark for the remainder of the protocol as much as possible.

26. Wash 1 × 5 minutes with WB.
27. Was 2 × 5 minutes with PBS.
28. Remove the PBS and mount the coverslips in polymount:
  a. Apply two drops of polymount per microscope slide (one for each of the duplicate coverslips per condition). Mark the slides accordingly.
  b. Pick up the coverslips with tweezers and tip the side onto a dry tissue to remove the remaining PBS.
  c. Put the coverslip with the cells facing down on top of a drop of polymount.

***Note:*** Prevent formation of air bubbles between the microscope slide and coverslip. You can apply light pressure on the mounted coverslip to remove possible air bubbles, but try to avoid breaking them.

29. Dry the mounted coverslips overnight at 18°C–22°C in the dark.

**Pause point:** Store up till 1 week at 4°C until imaging.

### Day 5—Imaging of CPDs and elongating RNAPII to visualize clearance

**Timing: 1–2 h**

Objective: Image the CPD signal and the RNAPII signal through fluorescence microscopy to quantify the clearance of RNAPII from the damaged areas of the nuclei.

30. Setup and Image fixed samples on a Zeiss AxioImager M2 widefield upright fluorescence microscope using a 63× PLAN APO oil-immersion objective (NA 1.4, M27,) and an Axiocam 705 camera.
  a. Place the microscopy slides underneath the microscope using the following motorized components: a luminous field stop system and a motorized z-drive with 25 nm step size to bring the plane into focus.
  b. Illuminate samples with an HXP 120 metal-halide lamp using a Zeiss Colibri 5/7 light source and detect fluorescence with the following filter sets: DAPI (excitation 350/50 nm, dichroic mirror 400 nm, emission 460/50 nm), Alexa Fluor 488 (excitation 470/40 nm, dichroic mirror 495 nm, emission 525/50 nm), Alexa Fluor 555 (excitation filter: 545/25 nm, dichroic mirror: 565 nm, emission filter: 605/70 nm), or Alexa Fluor 647 (excitation filter: 640/30 nm, dichroic mirror: 660 nm, emission filter: 690/50 nm).
    ***Note:*** While these specifics apply to our microscope system, any other fluorescence microscope with suitable filters will do.
  c. Use an image size of 2464 × 2056 (full chip; uncompressed) with a scaling of 0.087 μm × 0,087 μm (size scaled: 214,18 μm × 178,72 μm), a 14 bit digitizer and 1,1 binning.
  d. Set the depth of focus at 72 μm and collected the image at 1 fixed Z-position.
  e. Record images with ZEN 2012 software (blue edition, version 1.1.0.0) gaining 3 channel stacked.czi images.
    ***Note:*** Test images are available at https://git.lumc.nl/afjdegroot/rnapii-clearance/-/tree/main/Test_dataset_rep1.
  f. With a 63× objective on a widefield fluorescence microscope, bring the nuclei in the DAPI channel into focus, starting with a 0 h after UV condition (see step 31). Search for areas on the coverslip with nuclei that contain a bright CPD focus in the A488 channel. See Figure 1B for an example.
    ***Note:*** Avoid areas on the coverslip where many nuclei overlap to prevent measurement of fluorescence signal belonging to multiple cells.
31. Determine the optimal exposure time for RNAPII in the Alexa Fluor 555 channel by examining the 0 h post-UV coverslip sample and setting the exposure time as high as possible without causing overexposure.

***Note:*** The cells in the 0 h condition will have the highest RNAPII signal because the least amount of RNAPII will have run off the genes at the start of the DRB treatment.

32. Adjust the exposure time of the DAPI and the CPD channels so that the channel intensity reaches approximately 60% of the detector’s dynamic range.

***Note:*** This prevents both over- and under-exposure. Subsequently, keep the exposure times consistent per channel across all images and conditions within the experiment.

**CRITICAL:** Set the CPD exposure time leaving some range for higher CPD signal without overexposing, as the CPD signal can vary between different coverslips.

33. Image 50 to 100 cells with clear damages for each condition. Store the images in the appropriate format (for example Carl Zeiss Image Data Format, .czi files) and name each image according to its condition.

**CRITICAL:** Make sure each image is in focus and keep the set exposure times of all channels equal across all images and all conditions.

***Note:*** Name each image according to its condition, followed by a hyphen (−) and the image number. For the downstream data analysis it is convenient to avoid any additional hyphen (−) or period (.) symbols in the naming of each condition. An example of an appropriate image file name is “Wildtype_0 h-01”.

### Day 6—Quantification and statistical analysis

**Timing: 1–2 h**

Objective: Quantify the RNAPII signal inside and outside the UV-damaged areas from the microscope images of each time point to determine the level of RNAPII clearance over time.

**Figure 2:**
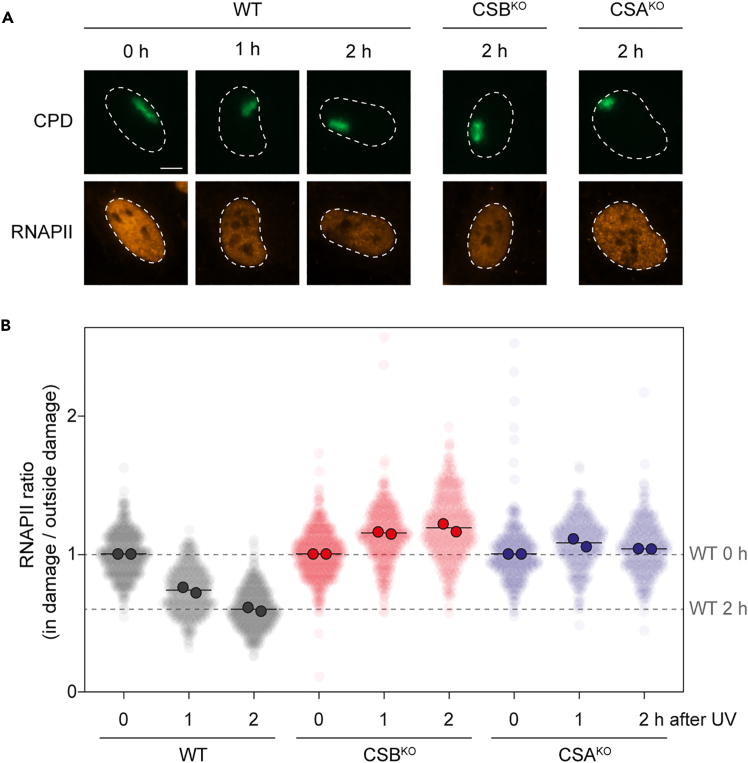
Expected RNAPII clearance outcome (A) Representative images showing the RNAPII clearance at 0, 1, and 2 hours after UVC-radiation. RPE1-hTERT (WT), RPE1 CSB-KO, and RPE1 CSA-KO cells are depicted with their respective IF signals for CPD (green), and RNAPII (orange) for each cell line. The dotted line marks the cell nucleus. The scale bar represents ∼5 μm. (B) Expected outcome of the RNAPII clearance protocol after quantification. The graph shows individual selected and quantified data points of two individual replicates/datasets per condition. RPE1-hTERT (WT), RPE1 CSB-KO, and RPE1 CSA-KO cells are depicted with their respective quantified value. All individual data-points are shown per time point as the ratio of the RNAPII signal inside the damaged area over the area outside, normalized to the 0 hour time point. The black line represents the average value of the means of the two independent experiments.

34. Run the script entitled “RNAPII_clearance_DataExtraction_ImageJ_DvdH_20250905_final.txt” in ImageJ to measure the RNAPII intensities and CPD intensities inside and outside the areas of local damage in the nuclei:
  **CRITICAL:** Use a computer with the minimal requirements for ImageJ (https://imagej.net/downloads.).
  **CRITICAL:** Only stacked images can be processed by the macro. In this protocol 3-channel stacked.czi files obtained in Zen (2012 Blue edition; Zeiss) were used.
  ***Note:*** A completed processing sheet (containing data from replicate 1 of the experiment presented in Figure 2) and an example test dataset from replicate 1 (including the first image of each condition and time point; 9 pictures) are available at https://git.lumc.nl/afjdegroot/rnapii-clearance.
  a. Open the script (https://git.lumc.nl/afjdegroot/rnapii-clearance).
  b. Select the folder containing the microscope images, then open the first image in that folder. The settings window will appear (consult also “Supplemental_manual_V2” on GitLab for visual and graphical support).
  c. Define the settings for the macro to measure the RNAPII signal in the A555 channel inside and outside the damaged areas (defined by CPD staining in the A488 channel) of the nuclei (defined by the DAPI channel).
    i. For RPE1 cells, an appropriate nucleus diameter to set is between 10 and 30 units.
      ***Note:*** Depending on the cell type and the image resolution, the optimal nucleus diameter setting for detecting individual nuclei may vary. The nucleus diameter can be determined by drawing a line across the nucleus using the Line Tool (https://imagej.net/ij/docs/tools.html) in ImageJ, before running the macro. To do this, open an image and draw a line across the diameter of a nucleus, then click Analyze > Measure. The measured value will appear in the 'Length' column, according to the defined unit size.
      ***Note:*** Although nuclei detection can be optimized, the exclusion of single nuclei and the occurrence of duplicate detections cannot be fully avoided, but they should be minimized as much as possible.
    ii. Set the number of channels to 3.
    iii. Set the nuclei channel number to 1 (DAPI) and the focus selection channel number to 2 (CPD staining in A488) (“Supplemental_manual_V2” page 1D). The measurement channel number to 3 (RNAPII staining in A555) is automatically set by the macro.
    iv. Set a minimal focus area of 0 units as a threshold for the minimal local CPD size.
      ***Note:*** Let the macro include all areas of high CPD signal. Too small damage areas can be excluded later in the analysis step in Excel.
      Set the threshold detection for local damages and nucleus detection to Yen and Li, respectively.^12^
      ***Note:*** The CPD intensities are required to exclude suboptimal damage areas from the analysis.
      ***Optional:*** The focus area threshold can be manually set to a specific value by clicking on “Threshold detection”>”Manual Input Below” and specifying the desired number of units.
    v. Press “OK” button to run the macro with these settings.
      ***Note:*** The macro will generate a folder entitled “_Results_focus analyses” in the folder containing the images. This folder contains an Excel document with the measured intensities of the regions of interest (ROIs), namely for each nucleus the damage ROI and the undamaged ROI. The ROIs are found in the compressed (zipped) folders in the same results folder, as well as a .txt document with the details of the settings of the macro run.
      ***Note:*** The macro measures all three channels simultaneously for each focus area, recording the intensities for each damaged ROI as well as the undamaged ROI. The data are then compiled sequentially into a single Excel table. Additionally, the macro directly measures the average background intensity for each channel in each image and reports it per focus area in the Excel table (see step 35).
35. Open the Excel file containing all focus area and background intensity data and define the column identities:
  a. Open the Excel table “_Results_focus analyses” containing the intensity data and background values for each channel.
  b. Define the data columns so that they can be copied into the datasheet (see step 36).
    i. Column A names the data point number, each row representing one cell (containing data for the damage ROI and the undamaged ROI in sequential columns for each channel).
    ii. Column B represents the label given to the data point.
    iii. Column C-I represents the data for channel 1 (DAPI) of the damage ROI (inside the focus area; overlaying the CPD damage spot).
    iv. Column J-N represents the data for channel 1 (DAPI) of the undamaged ROI (outside the focus area; overlaying the undamaged part of the nucleus).
    v. Column P-T represents the data for channel 2 (CPD) of the damage ROI.
    vi. Column U-Y represents the data for channel 2 (CPD) of the undamaged ROI.
    vii. Column AA-AE represents the data for channel 3 (RNAPII) of the damage ROI.
    viii. Column AF-AJ represents the data for channel 3 (RNAPII) of the undamaged ROI.
    ix. Column O, Z, and AK represents the average background signal data (per data point) for channel 1, 2 and 3, respectively.
36. Insert the collected data in the Excel template “DRB runoff local UV worksheet_v11_template” available at (https://git.lumc.nl/afjdegroot/rnapii-clearance).
  **CRITICAL:** The template performs the necessary calculations based on the measured data points and selection parameters; only fill in all the cells in yellow, as described in detail below. Make sure to use the “copy as plain text” function to avoid any unwanted changes in the sheet. A filled in example Excel sheet (with data from replicate 1 as depicted in Figure 2) is available at https://git.lumc.nl/afjdegroot/rnapii-clearance. Additionally, a graphical supplementary manual “Supplemental_manual_V2” of the macro and Excel sheet is available at https://git.lumc.nl/afjdegroot/rnapii-clearance.
  a. Open the “_Results_focus analyses” Excel file and copy column B (containing the labels of each ROI) to column C in the analysis template. Do not include the column titles in row 1 of the results file.
  b. Use the “text to columns” data tool to split the ROI names twice: first at the period (.) symbol and then at the hyphen (−) symbol, resulting in the condition labels in column C and the image numbers in column D. (see also Supplemental_manual_V2 page2E-G).
    ***Note:*** Let the second split paste the image numbers over everything that followed the period (.) symbol, as that information is not needed.
  c. Copy column AA (the ROI areas), of the RNAPII (channel 3) channel, from the results file to column G in the analysis template and column AB (the ROI intensity means) from the results file to column H in the analysis template. Exclude the column titles in row 1 of the results file.
  d. Copy column P (the ROI areas), of the CPD (channel 2) channel, from the results file to column F in the analysis template and column Q (the ROI intensity means) from the results file to column I in the analysis template.
  e. Copy column AF (the ROI areas outside the damaged area), of the RNAPII (channel 3) channel, from the results file to column K in the analysis template and column AG (the ROI intensity means) from the results file to column L in the analysis template. Exclude the column titles in row 1 of the results file.
  f. Copy column U (the ROI areas outside the damaged area), of the CPD (channel 2) channel, from the results file to column J in the analysis template and column V (the ROI intensity means) from the results file to column M in the analysis template.
  g. Copy column AK (average background signal in the RNAPII channel of each image) and paste them column N. Exclude the column titles in row 1 of the results file.
  h. Fill in the name of each condition in the preferred order in the yellow area starting at cell AI43.
    ***Note:*** The name should exactly match the condition names as they are in column C. You can use Excel’s “UNIQUE” function for this.
37. Use the graphs in the gray square to examine the individual data points in your dataset (“Supplemental_manual_V2” page 3K).
  ***Note:*** The top and bottom left graphs should show two populations ROIs: one with a small area and high CPD signal (the damaged areas), and one with a big area and low CPD signal (undamaged part of the nuclei). Include or exclude deviating data points by defining the threshold values in yellow (“Supplemental_manual_V2” page 3I):
  a. Using the top and bottom left graphs, define minimum and maximum sizes of the damaged areas in cells AG1 and AH1.
    ***Note:*** Exclude random small speckles in the CPD channel or very big CPD-positive regions that cover the majority of the nucleus area, which will be visible as outliers that deviate from the population of “damage” ROIs.
  b. Using the bottom right graph, define a maximum and minimum CPD signal intensity of the damaged ROIs in cells AI1 and AJ1.
    ***Note:*** Exclude extremely deviating CPD values to have comparable CPD intensities over all conditions.
  c. Adjust the cut-offs for focus size and CPD signal depending on the resolution and signal intensity of the original images.
    **CRITICAL:** Ensure that the CPD signal and focus size are filtered so that all conditions and time points have a comparable distribution, as illustrated in “Supplemental_manual_V2” page 3K.
38. The Excel sheet now calculates the ratio between the RNAPII signal inside and outside the damaged area for each nucleus and sort them in the correct condition in the table starting at column BW. These data are normalized to each 0 h time point in the table starting from column CY (example: “Supplemental_manual_V2” page 3L). The Excel table also generates a plot showing the average RNAPII signal per condition inside and outside the damaged area (column AT row 63). Additionally, Excel calculates summary data per condition and displays it in column AJ-AM.
  a. Plot the normalized (normalized to 0 h) data using PlotsOfData, available at https://huygens.science.uva.nl/PlotsOfData/(or other data-processing software), as shown in Figure 2B.

***Note:*** The RNAPII intensity ratio was normalized to the 0 h mock for each cell line to quantify UV-C-induced RNAPII clearance. Normalization was performed individually, as basal RNAPII levels vary between cells. This approach allows assessment of RNAPII clearance relative to the 0 h condition, rather than comparing absolute RNAPII levels between conditions. Unnormalized values can also be obtained from the Excel table if needed.

## Expected outcomes

### Quantification of RNAPII clearance in different conditions

During the three timepoints of 0 h, 1 h and 2 h after local UV irradiation, the RNAPII levels outside the damaged area slowly decrease because of the DRB treatment that causes RNAPII to run off genes, whereas the RNAPII levels inside the damaged area decrease more quickly because of the active removal by TCR. The ratio of RNAPII levels in the damaged and undamaged area (inside/outside RNAPII ratio) over time therefore reflects the ability of cells to clear RNAPII from damaged chromatin through TCR.

Results from this protocol for one cell line with efficient RNAPII clearance (RPE1 WT) and two cell lines with defective RNAPII clearance (CSB-KO can CSA-KO) are shown in Figures 2A and 2B. Directly after local UV irradiation, levels of transcribing RNAPII in the damaged area are still equal to the levels in the undamaged area in each condition. Therefore, when dividing the RNAPII signal inside over the signal outside the damaged area, this ratio is approximately 1 (usually slightly over 1). In RPE1 WT, a cell line that is fully TCR proficient, the inside/outside RNAPII ratio drops to approximately 0.4–0.5 at 2 h after local UV irradiation (Figures 2A and 2B). However, in cells that are deficient in RNAPII clearance, such as CSB- or CSA-deficient RPE1 cells, there is only the DRB-inflicted runoff, but no active RNAPII clearance from damages by TCR.^4^ Consequently, the inside/outside RNAPII ratio in these cells does not decrease over time (Figures 2A and 2B). To test whether the difference in RNAPII clearance is statistically significant, a one way ANOVA with Turkey or Dunnett post-hoc test can be performed comparing each time point of the tested conditions.

## Limitations

While this protocol is very insightful, accessible and applicable to many cell lines, it still has some limitations. For example, even when the steps are carefully followed, the quality of the locally damaged areas in the nucleus can vary. To increase the chance of having enough nuclei with a clear local damage, we recommend to include duplicates of every condition, as described in the protocol. The high UV dose used (100 J/m^2^) also results in a higher chance of more clear damages. Moreover, this high damage load, as well as the continuous DRB treatment during the time after UV, create experimental conditions that do not resemble physiological conditions. However, this experimental setup specifically allows monitoring the fate of the RNAPII molecules already elongating at the time of damage infliction.

## Troubleshooting

### Problem 1

The local damages created and later visualized by IF staining are not bright enough, too vague, too small or too big to be recognized by the macro (related to step 11). See also Figure 1B.

### Potential solution

If the micropore filter is not properly placed onto the coverslips during step 11, this can lead to low-quality damages. The most likely cause is that filters are too wet with PBS during the irradiation process. In these scenarios, UV-C light is blocked or scattered, resulting in vague CPD spots that might not be recognized as local damages by the macro. In addition, when a damaged areas is too large, it may cover the majority the nucleus, making it difficult to calculate the inside/outside RNAPII ratio accurately. Proper local damage spots are depicted in Figure 1B, showing round bright spots with sharp edges, covering less than 25% of the nucleus and ideally with approximately equal intensities between different cells and conditions. However, the following steps can be taken into account to ensure high-quality local damages.

• Apply the filters properly: ensure that the micropore filter fully covers both duplicate coverslips. Avoid moving the filter after mounting it on top of the coverslip, as this may distort the pores or displace the cells. Also, make sure not to place two filters on a single coverslip.
• Drops of PBS between the cells and the filter, or on top of the filter, will scatter and reduce UV irradiation. Remove any remaining PBS drops from the filter by gently touching the side of the filter with a tissue. After applying the filters to the coverslips, carefully wipe away any residual PBS from the top of the filters using the corner of a tissue.

### Problem 2

Although the majority of damages are of good quality, the brightness and size varies a lot over the different conditions (related to step 30–33, 37).

### Potential solution

Low quality local damages cannot be fully prevented during the irradiation step and should be excluded during the analysis.

• During imaging (step 30), only take pictures of nuclei that have a good quality damaged area, as comparable in intensity and size as possible between the different coverslips.
• During data analysis (step 37), set the upper and lower limit of the size and CPD intensity of the damages in such a way that deviating damages are excluded from analysis.

### Problem 3

Cells fail to adhere to glass coverslips during culture or microscopy staining (related to step 1).

### Potential solution

Some cell types may fail to attach on coverslips because glass itself is a poor substrate for adhesion. The following steps can help enhance cell adherence to the coverslips.

• Pre-coat coverslips with ECM proteins (fibronectin, laminin, collagen) to provide integrin ligands; sterilize coverslips before coating (before step 1).
• Use a charged polymer such as poly-L-lysine to promote electrostatic attachment, often combined with ECM for long-term stability (Before step 1).

### Problem 4

Cells detach from coverslips following local UV-C (related to step 11).

### Potential solution

Cells may detach from coverslips following UV-C irradiation if the cells are too confluent or if the micropore filter is removed with too much force.

• Highly confluent cell cultures are more easily detaching during local irradiation and removal of the filter. Therefore, make sure that cells are less than 80% confluent at the moment of irradiation.
• Add a drop of PBS on top of the filter before removing it very carefully from the coverslips. This will minimalize the chance of the cells sticking to the filter.

### Problem 5

No or duplicate cell data is generated during the image analysis or no cells are detected during the data processing (related to step 34).

### Potential solution

When the macro does not generate data during image analysis, this most often reflects a mismatch between the nuclear detection thresholds and the cell type currently used. In this protocol, the nuclear size is limited to 10–30 units (step 34) and optimized for RPE1 cells imaged on a Zeiss Axio Imager M2 widefield microscope with our specific microscope settings. If no nuclei are detected, the minimal threshold value is likely set too low, causing exclusion of nuclei in the DAPI channel (channel 1) with diameters smaller than 10 units. If multiple nuclei are identified as a single object, the maximal threshold value may be set too high, resulting in merging of adjacent nuclei. Another potential source of data loss is the setting of the minimal focus area in channel 2 (step 34). When this parameter is not set to zero, valid data points may be excluded automatically. In this protocol, the minimal focus area is set at zero, and invalid foci are removed manually during downstream processing in Excel. This strategy prevents data loss and preserves researcher control during data processing. Finally, absence of results can also arise from poor image quality. Low resolution, weak signal intensity, or insufficient focus may lead to misdetection by the macro. Although duplicate detections, undersized nuclei, or suboptimal focus cannot be entirely avoided, adjustment of nuclear size thresholds, focus area settings, and acquisition parameters might improve the robustness of the analysis.

• Use the line tool, as discussed in step 34, to measure the diameter of the nuclei in channel 1 (DAPI). Measure at least a few cells and multiple pictures to calculate an average. Subsequently, use the minimal and the maximal value (length in units) in the macro to set the threshold before running the macro (Supplemental_manual_V2 page1D).
• Set the focus area to 0 so that all the in-focus data points will be included (Supplemental_manual_V2 page1D). Subsequently, use the Excel sheet to filter the data (step 37).
• Change the minimal and maximal signal range on the widefield fluorescent microscope that is used and match this ratio in the macro text to prevent the loss of data (these values were in the macro; see RNAPII_clearance_DataExtraction_ImageJ_DvdH_20250905_final.txt) or increase the maximal signal intensity in the macro itself.

## Resource availability

### Lead contact

Further information and requests for resources and reagents should be directed to and will be fulfilled by the lead contact, Martijn S. Luijsterburg (s.m.luijsterburg@lumc.nl).

### Technical contact

Technical questions on executing this protocol should be directed to and will be answered by the technical contact, Bram A.F.J. de Groot (a.f.j.de_groot@lumc.nl), Paula van der Meer (p.j.van_der_meer@lumc.nl), Diana van den Heuvel (d.van_den_heuvel@lumc.nl).

### Materials availability

In this study no new materials or compounds were created.

### Data and code availability

All data and the code used for this study is available at https://git.lumc.nl/afjdegroot/rnapii-clearance. The following ImageJ packages were used during this study and can be found at https://imagej.net/ij/doi:10.1038/nmeth.2089 and https://www.openmicroscopy.org/bio-formats/downloads.

## Acknowledgments

This work was funded by the 10.13039/100010663European Research Council Consolidator Grant STOP-FIX-GO (grant agreement no. 101043815) and the Netherlands Scientific Organization (NWO) Vici grant (VI.C.212.005), both awarded to M.S.L. The funders had no role in the study design, the data collection and analysis, the decision to publish, or the preparation of the manuscript.

## Author contributions

B.A.F.J.d.G.: investigation, formal analysis, visualization, validation, and writing – original draft. P.J.v.d.M.: conceptualization, investigation, validation, writing, review, and editing. D.v.d.H.: conceptualization, software, writing, review, and editing. M.S.L.: conceptualization, funding acquisition, supervision, writing, review, and editing.

## Declaration of interests

The authors declare no competing interests.
